# Scaling up of physical activity interventions in Brazil: how partnerships and research evidence contributed to policy action

**DOI:** 10.1177/1757975913502368

**Published:** 2013-12

**Authors:** Diana C. Parra, Christine M. Hoehner, Pedro C. Hallal, Rodrigo S. Reis, Eduardo J. Simoes, Deborah C. Malta, Michael Pratt, Ross C. Brownson

**Affiliations:** 1. Washington University in St. Louis, Prevention Research Center, St. Louis, MO, USA; 2. Washington University in St. Louis, Division of Public Health Sciences and Alvin J. Siteman Cancer Center, School of Medicine St. Louis, MO, USA; 3. Federal University of Pelotas, Post-graduate Program in Epidemiology, Pelotas, Brazil; 4. Pontiff Catholic University of Parana, Department of Physical Education; Federal University of Parana, Department of Physical Education, Curitiba, Brazil; 5. University of Missouri, School of Medicine, Department of Health Management and Informatics, Columbia, MO, USA; 6. Ministry of Health of Brazil, Division of Situation Analysis and Prevention of Non-transmissible Diseases, Brasilia, Brazil; 7. Centers for Disease Control and Prevention, Office of Global Health, Atlanta, GA, USA

**Keywords:** physical activity, policy, practice, program planning, management, collaboration, partnership, communicable disease

## Abstract

The global health burden due to physical inactivity is enormous and growing. There is a need to consider new ways of generating evidence and to identify the role of government in promoting physical activity at the population level. In this paper, we summarize key findings from a large-scale cross-national collaboration to understand physical activity promotion in Brazil. We describe the main aspects of the partnership of Project GUIA (Guide for Useful Interventions for Activity in Brazil and Latin America) that sustained the collaborative effort for eight years and describe how the evidence gathered from the collaboration triggered political action in Brazil to scale up a physical activity intervention at the national level. Project GUIA is a cross-national multidisciplinary research partnership designed to understand and evaluate current efforts for physical activity promotion at the community level in Latin America. This example of scaling up is unprecedented for promoting health in the region and is an example that must be followed and evaluated.

## The problem

More than five million people die every year from a vast range of non-communicable diseases (NCDs) attributable to physical inactivity (1). According to the existing evidence, a wide range of interventions have been shown to be effective for changing population levels of physical activity (2); however, most evidence has been generated from high-income countries in Europe and North America (3,4). Only a handful of physical activity interventions have been evaluated in the context low and middle-income countries such as Brazil, generating important evidence that has guided the political and public health agenda of the country (5-7).

Documenting and filling gaps concerning physical activity interventions is an important step for addressing the NCD epidemic in low and middle-income countries for various reasons. First, the proportion of the world population residing in low and middle-income countries is larger (84%) than the proportion living in high-income countries, particularly those residing in urban areas (8). Second, NCDs are now superseding infectious diseases in many regions of the world, and an estimated 84% of the NCD global burden occurs in low- and middle-income countries (9). For instance in Brazil, 72% of all deaths in 2007 were attributable to NCDs (10). Similarly, the prevalence of physical inactivity among adults surpasses 40% in Brazil (11). The urgency of the NCD epidemic was reinforced by the recent United Nations General Assembly, which approved an unparalleled draft political declaration to rally the world towards addressing the growing NCD epidemic (12). The Assembly identified the promotion of physical activity as one of the main strategies to counteract the rising burden from NCDs. Third, the Latino population is increasing around the world, particularly in the United States, reaching 50.5 million people from Hispanic or Latino origin (13). Finally, Latin America is a region with vast social inequalities which have a direct impact on the health outcomes of its population, a situation requiring governmental action (14).

## Establishing a course of action

According to the traditional public health paradigm, the efficacy of interventions is first tested in small-scale randomized studies, and those proven to be successful are then regarded as the interventions that work. However, most of them never make it into practice to become large-scale public health interventions (15). We argue that for public health and physical activity promotion, a reverse and complementary approach may be preferable in many circumstances. Thus, if we are serious about changing physical activity levels of the world’s population, we should ‘get our hands dirty’ by evaluating interventions that have been running in the field for years; in other words, we should collect practice-based evidence (16-18). In Latin America, this includes evaluating on-going government-sponsored interventions that have been running without rigorous evaluations. There has been considerable debate about the role of governments in addressing key public health challenges, particularly those addressing lifestyle behaviors (19). We describe the characteristics of a cross-national academic–government partnership that led to the evaluation of a local government-sponsored physical activity intervention, which in turn contributed to the scaling up of this intervention to the national level in Latin America.

## A cross-national academic–government partnership to support practice-based research

Project GUIA (Guide for Useful Interventions for Activity in Brazil and Latin America) is a network of recognized academic and governmental institutions from the United States and Latin America, particularly from Brazil, that have partnered since 2005 to conduct scholarly, applied research and to build cross-national collaboration. Project GUIA was the first initiative of its kind to attempt to understand and generate evidence from Latin America concerning physical activity promotion (5). Conceived as a strategy to inform practice and policy through evidence-based interventions in Latin America, the project aims to build, strengthen and maintain cross-national networks, assess the current state of the evidence through systematic reviews focused on internal and external validity, understand gaps and areas of priority by implementing practice-based research, evaluate physical activity programs at the community level, and disseminate results while building capacity in the region.

**Figure 1.**
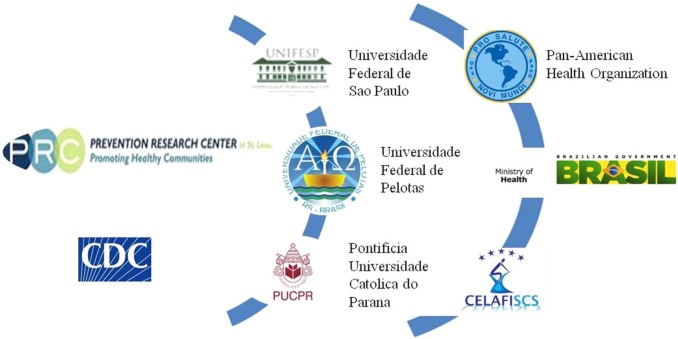
GUIA Core Institutions.

Funded by the Centers for Disease Control and Prevention (CDC) as a Special Interest Project (SIP), Project GUIA was a partnership between the following institutions: Prevention Research Center in St. Louis (PRC), Universidade federal do Sao Paulo (UNIFESP), Ministry of Health of Brazil, Universidade Federal de Pelotas (UFPel), Pontificia Universidade Católica do Parana (PUCPR), CELAFISCS, CDC, and Pan American Health Organization (PAHO). Some of these institutions played major coordinating and financial roles such as the PRC and the CDC, others were main research partners and played coordination roles in Brazil including UNIFESP, UFPel and PUCPR, while others were part of a larger advisory group including the Ministry of Health, PAHO and CELAFISCS (Figure 1). Although Project GUIA recently has been expanding networks and providing technical assistance to other countries from Latin America, including Colombia and Uruguay, the project is largely focused on Brazil. Some of the reasons for this selection include a great interest in the Universal Health Care model from Brazil, the vast economic, social and political influence of Brazil in the rest of Latin America, being the largest country and occupying 42% of the territory with more than 190 million people (20), a large proportion of Brazilians residing in the United States with over one million people (21), and the presence of already strong partnerships with government and academic institutions from Brazil. For instance, the Ministry of Health of Brazil and the CDC have a long history of collaborative work in various global health project and initiatives (22). In turn, the Ministry of Health from Brazil has strong partnerships with recognized academic and research institutions in the country in order to evaluate programs and implement interventions (23).

The project dedicated time and resources at the outset to identify what types of interventions were taking place in Latin America, which ones were regarded as effective, emerging or promising (24), and to define priorities for future evaluation. A systematic review of the evidence base for physical activity interventions in Latin America found sufficient evidence for recommending school-based physical education based on quality of execution and study design (24-26). The review also identified new intervention strategies such as physical activity classes in community settings and community-wide policies and planning that were selected for evaluation (6,27). The first intervention evaluated by Project GUIA was the Academia da Cidade (ACP) in Recife. ACP is an example of a program classified under the physical activity classes in community settings intervention category within the broad category of behavioral and social approaches for the promotion of physical activity. There are similar replications of ACP in other cities from Brazil including places like Aracaju, Vitoria and Belo Horizonte, which have also been evaluated with technical support from Project GUIA (28-31). There are additional examples and replications of similar programs in other countries from Latin America including Colombia and Chile (32,33). Project GUIA has recently completed an external validity evaluation of the ACP program in Recife and the Recreovia program in Colombia; results from this evaluation are currently in process for publication, and there is high potential for scale-up of similar programs. According to Project GUIA’s first systematic review, physical activity classes in community settings were one of the most frequent interventions reported in the literature, with 28 out of 42 selected studies (34). A subsequent update of the initial systematic review showed similar results (24), further supporting the selection of this program as a strong candidate for evaluation. Since 2002, this long-running institutionalized program has provided aerobic and dance classes taught by trained professionals, as well as organized walking groups and counseling on healthy lifestyles, in public spaces and free of charge to the population. The ACP seeks to coordinate actions of the Ministry of Health as well as state and municipal Health Departments, representing an innovative approach for physical activity promotion within the Universal Health Care System of Brazil. These programs seek to eliminate structural barriers for physical activity practice, such as lack of public spaces, by offering places where people can engage in guided physical activity.

Program and policy evaluations carried out by Project GUIA have used a mixed-methods approach (combined quantitative and qualitative methods) (31,35,36). The use of mixed methods has been increasingly recommended in light of the need to develop best practices for health promotion and the difficulty to generate evidence from randomized trials in physical activity and public health research (37,38). Logic models and historical evaluations of the program were developed a priori and were used to guide the quantitative and qualitative evaluations that followed. A qualitative study to assess barriers and facilitators of the programs by addressing the perception of users and non-users was carried out (39). A population-wide telephone survey found three factors that were associated with high levels of physical activity during leisure time: awareness of ACP, residing near an ACP site, and former and current participation in the program. In addition, a corresponding evaluation using systematic observation showed that more people were observed practicing moderate and vigorous physical activity in park sites where ACP takes place compared with matched control parks (40). An important finding from this evaluation is that more women and older adults were observed in ACP sites compared with non-ACP sites, and also more women than men were seen practicing vigorous physical activity in sites that offered the ACP. This observation provided evidence that these types of programs may be useful in reducing disparities in physical activity among high-risk population groups such as women, older adults and low socio-economic status (SES) populations.

## Scaling up of physical activity interventions in Brazil

Results from the evaluations carried out by Project GUIA, particularly the evaluation of the ACP as well as the parallel evaluations of the interventions in Aracaju, Vitoria and Belo Horizonte (28-31), were instrumental in the decision of the Brazilian government to expand the ACP to the entire state of Pernambuco in its 184 municipalities, and later to the entire country. In April 2011, the Brazilian Ministry of Health supported the national expansion of ACP under the name Academia da Saúde, to over 4,000 cities in Brazil (41). This is one of the largest scale-ups of a chronic disease prevention initiative taking place anywhere in the world, and provides an ideal natural experiment for evaluation of what works to promote physical activity. With the support of Project GUIA, the Ministry of Health of Brazil is developing a baseline evaluation of the program. It is hoped that findings can be used to develop future recommendations for scaling up public health interventions (15,42). The important role that the cross-national academia–government partnership of Project GUIA played in the scaling up of ACP can be seen in the following quote provided by the current Minister of Health of Brazil, Doctor Alexandre Padilha:

The Academy of Health program (Academia da Saúde) was launched by the Brazilian Ministry of Health in April 7th 2011. It foresees the construction of physical spaces with infrastructure, equipment and qualified human resources to give orientation to the population regarding the practice of physical activity. By 2015, 4000 units will be built in partnership with the municipalities. The Program is articulated with the primary health care system and it is focused on reducing structural barriers to the access to physical activity practice and healthy habits, particularly among low-income populations, thus reducing health inequities. This program is anchored in the on-going experiences in Brazil with evidence of effectiveness in increasing the frequency of physical activity of the population in Recife, Curitiba, Aracaju, Pernambuco and others, which were evaluated by the Ministry of Health in partnership with the Project GUIA and Brazilian Universities. This international partnership has provided opportunities for the adaptation and development of innovative assessment methodologies, showing that it is possible to create strong partnerships between academic institutions and governments. Also, such partnerships can assist in scientific development and in public health practice, supporting the decision-making process. Evidence of effectiveness generated by these evaluations was very important to guide the creation of the Academy of Health program for the entire population of Brazil.

Just like the ACP, the Academia da Saúde program will be implemented in 4000 municipalities in the form of ‘polos’ (places where the physical activity classes take place). The program will be implemented in partnership with the municipalities, providing physical spaces endowed with infrastructure, equipment and qualified human resources for physical activity guidance during leisure time as well as screening and counseling on healthy lifestyles. The government of Brazil has identified physical activity as a key strategy for the prevention of NCDs and is also interested in helping reduce health and social inequalities through the empowerment of the community and the individual (43). One of the main goals of programs such as the ACP and the Academia da Saúde is to provide a viable outlet for physical activity, by developing infrastructure and offering professional orientation without the need to enroll in fee-based, private facilities such as gyms and other sports clubs. Results from the evaluation of ACP programs also showed evidence of a higher likelihood of use by high-risk populations for physical inactivity including women, older adults, and low SES populations (40). In this sense, investing in a national program for the promotion of physical activity seems to be a good use of resources and political will, given the large social and health inequities of the Brazilian population.

There have been several national policies and decrees that have facilitated the implementation and evaluation of physical activity programs in Brazil and have provided the right environment for collaborations between the academic and the government sectors. Some of these events include the creation of Brazil’s national policy of health promotion, approved by decree in 2006 (44), preceded by the decree 2608 from 2005 which for the first time provided funds to municipalities specifically geared towards health promoting strategies aiming to increase physical activity (45). The creation of a national network for physical activity promotion in Brazil facilitated the adoption and dissemination of new methodologies for implementation and evaluation of physical activity programs (23). In August 2011 the Ministry of Health launched the strategic action plan for combating NCDs (41) in which the Academia da Saúde program plays a central role for the promotion of physical activity and the prevention of chronic disease at the national level.

Project GUIA and the evaluation and implementation efforts from the Brazilian Ministry of Health have created a unique and unprecedented opportunity to evaluate the feasibility of scaling up interventions, a growing research area of interest that is gaining recognition due to its potential for improving population health in a cost-effective way (15,42). There are a few recognized examples of countries that have scaled up public health initiatives to increase population levels of physical activity (46), but few if any examples have emerged from other regions of the world and, in particular, from Latin America. Brazil joins this list of countries by launching the Academia da Saúde program.

## Lessons learned to inform future global cross-sector collaborations

To evaluate the progress of Project GUIA, an anonymous online survey with open-ended responses was conducted with 21 project members (57.1% were from Brazil and 42.9% from the United States). Identified through content analysis, the main factors responsible for Project GUIA’s success were: (1) a national and international network of researchers with strong leadership, experience, passion, and collaborative spirit; (2) collaboration between senior and young researchers; (3) publishing in high-impact scientific journals; (4) well-established and rigorous methodology; (5) political support and partnership between academic and government institutions; (6) dissemination efforts; and (7) regular in-person meetings and constant follow-up of project activities. The survey also identified some ways in which the project could be improved, including: (1) expand to rest of the Americas; (2) enroll all members of Project GUIA in the decision-making process including research priorities; (3) increase opportunities to meet and work in person; (4) increase opportunities for student, staff, and researchers’ exchanges; (5) develop more connections with other regions from Brazil and increase participation of societies and other governmental and non-profit organizations; (6) closer monitoring of the productivity of each staff member, research assistants and researchers from Project GUIA; and (7) develop multiple funding streams to guarantee continuity and flexibility. Many of the lessons learned from the experience of Project GUIA could be applicable to similar projects or initiatives in other nations.

When trying to understand the successes that the partnerships of Project GUIA have had since its implementation, it is important to keep in mind that the project arrived at a time where public health agencies from Brazil were ready to embrace NCD prevention initiatives. In addition, the sustained funding from CDC and the inter-disciplinary nature of the research and evaluation facilitated the efforts initiated. Regardless, it is important to highlight a few of the factors through which we found a recipe for success with an academia–government partnership in a multi-country research collaborative initiative:

1. Country and academic institution offering/suggesting partnership has established/funded academic–government partnership;
2. Recipient country and academic institution(s) with a structured collaborative partnership (Ministry of Health of Brazil has established renewable contracts of work with a network of university partners in the fields of assessment, evaluation and research);
3. Recipient country had a government-established leadership on health promotion and disease prevention initiatives;
4. Public health ties and collaborations in health promotion and disease prevention between offering and recipient country governments already existed before the project started – some of the same actors/players involved in the past also participated in current projects and efforts for collaboration;
5. Identifying and inviting diverse partners in academia (all sizes and geographically diverse universities), government (federal, state and local) and non-governmental (PAHO, CELAFISC, others) in the recipient country;
6. Cultural sensitivity approach, always allowing recipient partners (government, academia and other partners) to initiate process of identifying or prioritizing research efforts according to local context;
7. Begin by assessing/reviewing knowledge base – assuming nothing or very little is known in order to identify research needs and disseminate findings;
8. Implement assessment/review that is comprehensive enough to identify existing practice-based evidence, not only what is ‘scientifically sound’;
9. Implement practice-based orientation to evaluate ‘best possible’ evidence, with special emphasis on main partner’s funded projects;
10. Be ready to change and adapt course of action to address identified needs of partners.

## Conclusion

The story behind the national initiatives for physical activity promotion in Brazil and Project GUIA combines commitment, passion, leadership, partnerships and science around public health. Moreover, the political, financial, and social environment created favorable conditions for fostering these assets that benefited physical activity and chronic disease prevention in Brazil. Project GUIA played a part in this story by partnering with the Brazilian Ministry of Health and leading Brazilian researchers to contribute practice-based evidence that informed national policy. The partnerships created a unique synergy that resulted in exceptional research productivity with direct linkages to public health practice. It is hoped that the confluence of events that contributed to scaling up physical activity promotion in Brazil will not be a rare situation, but one that can stimulate a ripple effect and be replicated to improve the health of populations living in other low and middle-income countries.

**Key messages:**

1. Global efforts to promote physical activity need public health stewardship with a central role for government, civil society, and other sectors.
2. Research efforts and practice-based evidence can lead to governmental action and policy change, thus improving the health of the population.
3. Lessons from cross-national partnerships such as Project GUIA can be used to inform global efforts to enhance the likelihood of success.
